# Medial femoral condyle restoration technique in total knee arthroplasty

**DOI:** 10.1186/s42836-025-00353-8

**Published:** 2026-03-11

**Authors:** Quanbo Ji, Yan Wang, Lin Hao, Yang Luo, Peng Ren, Ming Ni, Lei Geng, Guoqiang Zhang

**Affiliations:** Department of Orthopaedics, General Hospital of Chinese People’s Liberation Army, Beijing, 100853 China

**Keywords:** Total knee arthroplasty, Medial femoral condyle restoration, Soft tissue release

## Abstract

**Objective:**

To introduce a medial femoral condyle restoration (MFCR) technique for total knee arthroplasty (TKA) and compare its clinical outcomes with conventional mechanical alignment (MA) in varus osteoarthritis.

**Methods:**

In this prospective randomized trial, 126 consecutive patients with varus osteoarthritis undergoing TKA (January 2021–January 2023) were assigned to MFCR or MA. MFCR surgical key points were medially focused quantitative compensation of cartilage loss by reducing distal/posterior medial femoral resections using thickness-specific shims (0.5–2.0 mm, 0.5-mm steps) guided by Outerbridge grading and calibrated probing. Participants were randomized to receive either the MFCR technique or the conventional MA technique. Intraoperative outcomes (blood loss, operative time, hospital stay, and medial release) and postoperative ROM were recorded; functional outcomes included WOMAC and walking VAS pain. Continuous variables were expressed as mean ± standard deviation and analyzed using one-way analysis of variance.

**Results:**

The mean age of the MFCR group and control group was 68.3 ± 7.4 years and 67.9 ± 8.7 years, respectively (*P* = 0.4236). Preoperatively, the mean WOMAC score of the groups was 67.2 ± 9.8 and 62.3 ± 16.4, respectively (*P* = 0.2524). The mean varus knee angle was 18.2° ± 7.2° and 17.3° ± 8.9°, respectively (*P* = 0.6735). The mean time for soft tissue balancing was 5.1 ± 2.6 min and 12.1 ± 4.3 min in the MFCR and control group, respectively (*P* = 0.017). The mean operative time was 50.6 ± 12.1 min and 58.9 ± 13.8 min in the MFCR and control group, respectively (*P* = 0.011). The mean hospital stay time was 1.8 ± 0.7 days and 3.2 ± 0.9 days in the MFCR and control group, respectively (*P* = 0.028). At 2 years postoperatively, the WOMAC scores were 29.9 ± 17.9 and 43.6 ± 13.7, respectively (*P* = 0.0325). Postoperative nausea/vomiting occurred less frequently in the MFCR group (*P* = 0.0391), with no other complications observed during follow-up.

**Conclusion:**

MFCR restored the anatomy of the medial femoral condyle by quantitatively preserving medial femoral bone to compensate for cartilage loss within a bony-first, minimal-release workflow. Compared with MA, MFCR reduced perioperative burden and improved early function, and can be implemented using a simple, reproducible technique without advanced imaging or robotics.

Video Abstract

**Supplementary Information:**

The online version contains supplementary material available at 10.1186/s42836-025-00353-8.

## Introduction

Total knee arthroplasty has been an effective treatment for advanced knee osteoarthritis [1]. Despite TKA’s established efficacy in pain reduction and functional improvement, patient satisfaction surveys reveal persistent dissatisfaction regarding high-demand functional recovery [2–4]. There are many factors that contribute to patient dissatisfaction [5–7]. Medial instability, including mid-flexion instability, is an important one because it may result in abnormal kinematics [8, 9] and poor functional outcomes, leading to patient dissatisfaction.

Normal knee kinematics is characterized by “medial stable” and “medial pivot” [10–12]. Medial pivot refers to a knee-kinematic pattern in which the medial tibiofemoral compartment functions as a relatively stable center of rotation through flexion–extension, while the lateral compartment exhibits greater posterior rollback and translation. In TKA, reproducing a medial pivot entails prioritizing medial gap stability with controlled lateral laxity to approximate native knee biomechanics. One of the important factors that contributes to medial stability, especially in the mid-flexion, is the articular surface of the medial femoral condyle [13]. It is also known as the distal joint line and posterior condyle offset (PCO) [14]. Failing to restore the anatomy of the medial condyle (joint line elevation or PCO decrease) after total knee arthroplasty may disturb the spatial relationship between the femoral insertion site of the MCL and the medial femoral joint line, resulting in mid-flexion instability [15–17]. The PCO could not be fully restored, even using the current universal posterior referenced sizer.

The other essential structure that contributes to medial stability is the medial soft tissue, mainly the superficial MCL, which is characterized as static and isometric [18, 19]. Unfortunately, in the mechanical alignment (MA) technique, medial soft tissue release is a common procedure in the varus knee to correct the varus deformity in the meanwhile achieving a balanced rectangular gap [20–22]. These procedures may cause pathological lengthening or destruction of the normal MCL, resulting in loss of knee proprioception and MCL incompetence, leading to poor outcomes. Besides, studies have found that the coronal alignment of the lower limb varies and constitutional varus exists in the normal population and knee osteoarthritis patients [23–25]. The target of 0-degree alignment and perpendicular bone cut may not be physiological. In recent years, Dr. Howell introduced the kinematic alignment (KA) philosophy [26, 27]. Though there are advantages of KA in restoring the joint line orientation, concerns still exist about the extreme implant position, reproducibility with the manual instrument, only one implant designed for MA available, and long-term survivorship.

Building on the principles of KA, we introduce a novel medial femoral condyle restoration (MFCR) technique for TKA. The core of this technique is to restore the morphology of the femoral medial condyle by the meticulous bone cut of the distal and posterior medial femoral condyle with compensation for the cartilage loss. We also do some adjustments to the bone cut orientation according to the patient’s individual bone morphology in order to keep the limb alignment in a more physiological status, as well as reduce the soft tissue release. The purpose of this study was to compare the early outcomes of the MFCR technique with the conventional MA technique in TKA.

## Materials and methods

### Study design and participants

This study was approved by the Ethics Review Board at Chinese PLA General Hospital. Consecutive patients with primary varus osteoarthritis scheduled for primary TKA between January 2021 and January 2023 were screened. Key exclusions were valgus knees, varus > 20°, flexion contracture > 15°, extra-articular deformity, inflammatory or traumatic arthritis, ligament insufficiency/instability, active infection, severe neurovascular disease, and inability to adhere to follow-up. Written informed consent was obtained from all participants.

Patients were randomized 1:1 to MFCR or MA using a computer-generated sequence (R software), with permuted variable block sizes of 4 and 6 to minimize predictability. The sequence was created by a study statistician who had no role in screening, enrollment, surgery, or outcome assessment. Allocation concealment was ensured with sequentially numbered, opaque, sealed, tamper-evident envelopes (SNOSE) prepared off-site; envelopes were opened after anesthesia induction by a research nurse not involved in outcome assessment.

Surgeons could not be blinded due to the nature of the procedures. However, participants were not informed of their allocation during hospitalization and follow-up; clinical assessors and radiographic reviewers were blinded to group assignment. Two independent blinded observers performed radiographic measurements; inter-rater reliability (ICC) is reported in the Results. Data were cleaned and analyzed by a statistician blinded to group coding until database lock.

The trial was prospectively powered for the continuous endpoint time, a direct surrogate of the study mechanism. Assuming a clinically meaningful between-group difference, two-sided *α* = 0.05 and 80% power. Allowing for attrition/non-adherence, we ultimately enrolled 126 patients, maintaining adequate power for secondary endpoints. Analyses followed a prespecified intention-to-treat framework with per-protocol sensitivity analyses.

All procedures were performed by the same arthroplasty team. Implant choice followed preoperative planning and intraoperative gap assessment. Rehabilitation pathways, anesthesia protocols, perioperative analgesia, thromboprophylaxis, antibiotics, and discharge criteria were standardized across groups.

### Surgical technique

#### Step 1: Exposure

All patients were under general anesthesia. The tourniquet was inflated before the skin incision. A standard parapatellar approach was used. A small triangle of anteromedial soft tissue, the deep layer of the medial collateral ligament, is detached as preliminary exposure. All osteophytes at the tibial plateau, the femoral condyle, and the intercondylar notch were then removed. If the flexion contracture existed, it was necessary to detach the adhesion between the posteromedial capsule and the tibia plateau and then remove the posteromedial tibial osteophytes.

#### Step 2: Medial femoral restoration technique

The tibial cut angle in the coronal plane was set based on the patient’s individual mMPTA. The tibia was cut in a 0–1-degree varus while the mMPTA was between 90° and 87°, in a 1–2-degree varus while the mMPTA was between 86° and 83°, and in the extreme case of mMPTA less than 83°, 2–3-degree varus cut can be performed. The tibial slope was set between 0° to 5°. We first set the extramedullary rod parallel to the tibial shaft as a baseline of zero degrees. It was adjusted by shifting the distal end of the extramedullary rod. Every 5–7 mm of lateral shift may increase 1 degree of varus. The thickness of the tibial cut refers to the unworn lateral plateau, to is the thickness of the thinnest liner, while the tibial cut at 2° or 3° varus. A 1–2 mm more bone should be added if the tibial varus cut is 0–1° to compensate for the reduced femoral resection.

The distal and posterior surfaces of the medial femoral condyle were visually graded using the Outerbridge classification. A calibrated ball-tip probe and feeler gauges (0.5 mm increments) were used to estimate the depth of cartilage loss relative to adjacent intact cartilage or sclerotic bone. To ensure reproducibility across cases, we prospectively adopted a simple conversion that guided how much bone to preserve to compensate for cartilage loss: Grade 0–1: 0.0 mm compensation, Grade 2: + 0.5 mm, Grade 3: + 1.0 mm, Grade 4 (near/full-thickness loss): + 2.0 mm. These values were chosen a priori as a pragmatic, easy-to-apply range that matches the commonly observed defect depths in varus knees and aligns with the surgeon’s long-standing practice patterns.

**Fig. 1:**
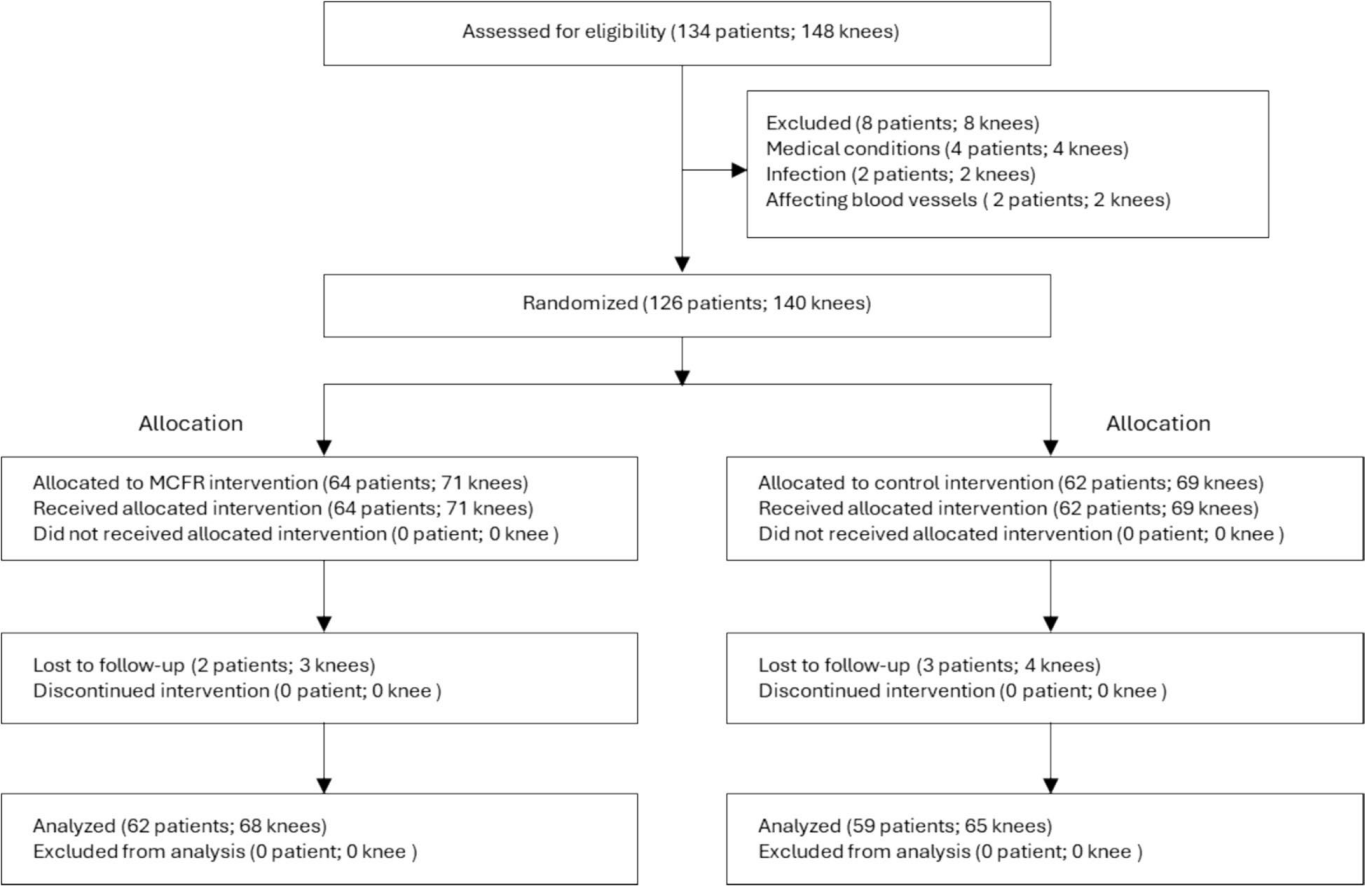
Clinical Application of the Medial Femoral Condyle Restoration Technique in Total Knee Arthroplasty. The surgical procedure involves the following steps: (**A**) Distal femoral osteotomy is performed with adjustment for medial condylar cartilage wear to preserve appropriate thickness; (**B**) A custom-developed device enables controlled femoral external rotation osteotomy with angle personalization; (**C**) Posterior condylar resection incorporates thickness-specific spacers to compensate for cartilage defects; (**D**) Final osteotomy is conducted after confirming femoral rotation and posterior condylar alignment; (**E–F**) A proprietary measurement device (anterior and anteroposterior views) ensures precision in determining femoral rotation and implant sizing, completing the anatomical restoration process in total knee arthroplasty

The distal cut on the medial condyle was reduced by the compensation derived above. Practically, the intramedullary distal cutting guide was seated on the distal medial condyle (Fig. 1), and bone was removed to elevate the medial cut plane by the intended amount (e.g., + 1.0 mm for Grade 3, + 2.0 mm for Grade 4). This achieved the stated “reserve 1–2 mm” when wear was advanced. Posterior condylar resection incorporated the same thickness-specific shims to reduce the posterior resection by the intended compensation (0.5–2.0 mm). The selection of shim thickness was based on the visual depth of the posterior defect, corroborated by the calibrated probe. For focal full-thickness posterior wear, a 1.5–2.0 mm shim was typically used; for partial-thickness wear, a 0.5–1.0 mm. For quality control, daily kerf calibration on a test block; total-removal cross-check (bone chip + kerf) vs plan, tolerance ± 0.5 mm. Gap verification with 0.5 mm feeler gauges/laminar spreaders.

The MCL subperiosteal elevation more than 2 cm below the joint line, MCL pie-crusting, PMC release, and SM detachment were recorded during surgery. Steps of the medial dissection 0.5–1 cm below the tibial plateau during exposure and removal of the osteophytes did not damage the normal soft tissue structure and were not recorded in the soft tissue release procedures in the study. We chased a normal lateral laxity in the MFCR technique. No more than 3 mm of a larger lateral gap than the medial gap under the varus/valgus stress test was accepted. If the medial side was tight both in flexion and extension, the tibial cut was checked, and a slight tibial varus cut could be added within 3° as the boundary. The mismatch of the flexion–extension gap could be balanced by adjusting the tibial slope. If there was still an imbalance after bony adjustment methods, further medial soft tissue release could be performed.

#### Step 3

Legion PS prostheses were used during surgery. There were standard and narrow versions of the legion femoral component available to match the patient’s individual anterior–posterior/medial–lateral dimension. After the bone cement had solidified, the tourniquet was deflated, and bleeding was electrically coagulated. Local infiltration was used for analgesia. the incision was closed without drainage.

#### Step 4

Cephalosporin was intravenously administered half an hour before surgery. A total of three doses were used until 24 h after surgery to prevent infection. Low molecular weight heparin was administered subcutaneously from post-op day 1 for VTE prophylaxis. Diclofenac sodium, acetaminophen, and oxycodone tablets were orally administered for analgesia.

#### Step 5

A gradual, step-by-step fast recovery protocol was implemented for all the patients. The patient was checked and instructed to perform straight leg raises and active knee flexion 6 h after surgery. On post-op day 1, the patient was allowed to get out of bed with a walker. After that, the patient was encouraged to walk without the walker. Once the patient could walk 20 m without any aids, the patient was allowed to go up and down the stairs. The patient was discharged after the surgery and went on rehabilitation as prescribed by the doctor. The patients were followed up 2 years after surgery.

### Data collection

The surgical time, intraoperative blood loss, and complications were recorded. Complications were defined as deep vein thrombosis, pulmonary embolism, calf muscular venous thrombosis, deep periprosthetic infection, wound complications, periprosthetic fracture, PONV, 30-day mortality, and 90-day readmission.

### Outcome evaluation

Assessments were conducted by a senior orthopedic surgeon with extensive knee surgery experience, who did not participate in treatments to reduce bias. Data were collected preoperatively and postoperatively at 2 years, via in-person interviews (for exams and questionnaires) or telephone (for patients unable to attend), ensuring consistent 2-year follow-up.

### Varus knee deformity

For evaluating varus knee deformity, frontal X-rays (acquired in varus/valgus postures) were used to measure the angle created by the crossing of the femoral and tibial mechanical axes. Specifically, the femoral mechanical axis was demarcated as a line extending from the midpoint of the femoral head to the midpoint of the femoral intercondylar notch, and the tibial mechanical axis was defined as the line from the center of the tibial plateau to the apex of the medial malleolus. This approach, aligned with conventional measurement standards in prior studies, allowed for consistent and accurate quantification of the varus deformity angle.

### Soft tissue release time

The duration for medial soft tissue balancing was defined as the interval from the initiation of the spacer test to the completion of the balance test. Specifically, the release times for individual medial soft tissue structures, including the medial collateral ligament, posteromedial corner, and semimembranosus tendon, were meticulously recorded. This protocol ensured precise documentation of the time required for targeted soft tissue interventions during the surgical procedure.

### WOMAC score

Clinical outcomes following total knee arthroplasty were evaluated using the Western Ontario and McMaster Universities Osteoarthritis Index (WOMAC) score. This psychometrically validated instrument comprises three core domains: pain (assessing severity during rest, locomotion, and weight-bearing activities), stiffness (quantifying morning stiffness duration and general stiffness levels), and physical function (evaluating performance in tasks such as ambulation, stair climbing, and flexion movements). Via administration of standardized questionnaires, the WOMAC score enables quantitative measurement, thereby providing a comprehensive and objective assessment of post-operative knee function and patient-reported outcomes.

### Walking VAS score

The Visual Analog Scale (VAS) for walking was utilized to evaluate pain intensity during ambulation. Patients were instructed to mark their perceived pain on a 100-mm horizontal line, where 0 mm corresponded to “no pain” and 100 mm to “the worst pain imaginable”. The assessment was conducted under standardized conditions, specifically during a 10-m walk test at a self-selected pace. This approach ensured consistent measurement across all participants, allowing for objective quantification of pain experienced during functional weight-bearing activities.

### Data analysis

For quantitative variables, symmetric distributions were described using mean and standard deviation, while asymmetric ones were characterized by median and interquartile range. One-way analysis of variance was employed to identify significant disparities between groups. Statistical significance was defined as a *P*-value < 0.05. All data were analyzed with SPSS software (Version 25.0; SPSS, Inc., Chicago, Illinois).

## Results

### Follow-up

**Fig. 2:**
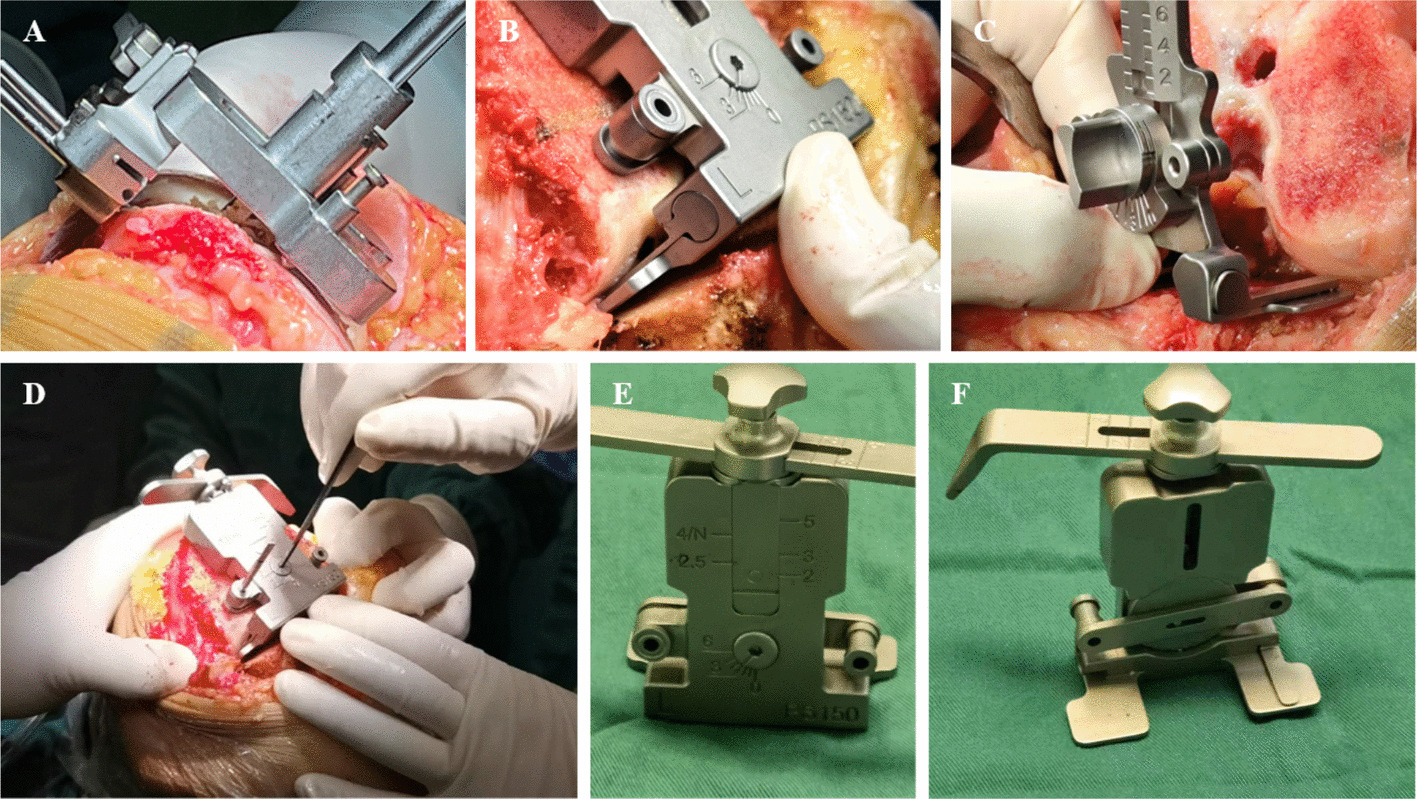
Flowchart of patient selection for the study. Eligibility assessment, exclusions, randomization into MFCR and control groups, follow-up losses, and final analysis are shown. Initially, 134 patients (148 knees) underwent eligibility assessment. Subsequently, 8 patients (8 knees) were excluded due to medical conditions, infection, and vascular involvement. A total of 126 patients (140 knees) were then randomized into two groups: the MFCR intervention group (64 patients, 71 knees) and the control intervention group (62 patients, 69 knees). During follow-up, 2 patients (3 knees) in the MFCR group and 3 patients (4 knees) in the control group were lost to follow-up. Ultimately, 62 patients (68 knees) in the MFCR group and 59 patients (65 knees) in the control group were included in the final analysis

A total of 134 patients (involving 148 knees) were assessed for eligibility. Among these, 8 patients (8 knees) were excluded, with the reasons for exclusion being as follows: 4 patients (4 knees) had specific medical conditions, 2 patients (2 knees) suffered from infection, and another 2 patients (2 knees) had conditions affecting blood vessels. Subsequently, the remaining 126 patients (140 knees) were randomized. They were divided into two groups: one group was allocated to the MFCR intervention, consisting of 64 patients (71 knees), all of whom received the assigned intervention. During the follow-up period, 2 patients (3 knees) were lost to follow-up, leading to 62 patients (68 knees) being included in the final analysis for this group. The other group was allocated to the control intervention, including 62 patients (69 knees). During follow-up, 3 patients (4 knees) were lost to follow-up, resulting in 59 patients (65 knees) being analyzed in the final analysis for this group (Fig. 2). The 2 groups did not differ significantly with respect to baseline characteristics (Table 1).

**Table 1:** Demographic and clinical data

	**MFCR (*N* = 62)**	**Control (*N* = 59)**	***P-*Value**
Age (yr)	68.3 ± 7.4	67.9 ± 8.7	0.4236
Sex, male/female	19/43	22/37	0.4403
Site, left/right	28/34	32/27	0.3182
BMI (kg/m^2^)	25.5 ± 6.2	26.1 ± 5.4	0.5629
Varus deformity (°)	18.2 ± 7.2	17.3 ± 8.9	0.6735
**Patellofemoral arthritis at the medial facet, KL grading (no. [%])**			
Grade 0	1 (1.61)	2 (3.39)	0.5206
Grade 1	15 (24.19)	17 (28.81)	0.5646
Grade 2	39 (62.90)	31 (52.54)	0.2486
Grade 3	7 (11.29)	9 (15.25)	0.5200
**Patellofemoral arthritis at the lateral facet, KL grading (no. [%])**			
Grade 0	22 (35.48)	28 (47.45)	0.1812
Grade 1	40 (64.51)	31 (52.54)	0.1812
**Outerbridge classification (no. [%])**			
Grade 0	1 (1.61)	2 (3.39)	0.5298
Grade 1	12 (19.35)	14 (23.73)	0.8934
Grade 2	19 (30.65)	16 (27.12)	0.6689
Grade 3	26 (41.94)	21 (35.59)	0.4743
Grade 4	4 (6.45)	6 (10.17)	0.4578
**ASA classification (no. [%])**			
I	27 (43.55)	31 (52.54)	0.3222
II	35 (56.45)	28 (47.46)	0.3222
**Quadriceps muscle strength, MRC grading (no. [%])**			
Grade 5	29 (46.77)	33 (55.93)	0.3138
Grade 4	33 (53.23)	26 (44.07)	0.3138
Range of motion (deg)	85.4 ± 8.9	87.3 ± 7.1	0.6289
**Specific comorbidities (no. of patients)**			
Hypertension	25 (40.32)	18 (30.51)	0.2596
Diabetes	10 (16.13)	6 (10.17)	0.3334
Cardiac disease	3 (4.84)	2 (3.39)	0.6890
Kidney disease	0 (0.00)	1 (1.69)	0.3033
Pulmonary disease	2 (3.23)	3 (5.08)	0.6206
Liver disease	2 (3.23)	1 (1.69)	0.5979
Cerebrovascular event	3 (4.84)	2 (3.39)	0.6890
Thyroid disease	2 (3.23)	1 (1.69)	0.5883

*BMI* = *body mass index, ASA* = *American Society of Anesthesiologists, KL* = *Kellgren-Lawrence, MRC* = *Medical Research Council. P-values of* < *0.05 were considered significant*

### General results

General results are shown in Table 2. The MFCR group (*N* = 62) had an intraoperative blood loss of 96.8 ± 21.3 mL, which was significantly lower than that of the control group (*N* = 59) at 109.4 ± 33.1 mL (*P* = 0.023). Similarly, the operative time in the MFCR group was shorter (50.6 ± 12.1 min) compared with the control group (58.9 ± 13.8 min), with a statistically significant difference (*P* = 0.011).

**Table 2:** Comparison of Clinical Outcome between the two groups

	**MFCR (*N* = 62)**	**Control (*N* = 59)**	***P*-Value**
Intraoperative blood loss (mL)	96.8 ± 21.3	109.4 ± 33.1	0.023
Operative time (min)	50.6 ± 12.1	58.9 ± 13.8	0.011
Medial release time (min)	5.1 ± 2.6	12.1 ± 4.3	0.017
Hospital stay (day)	1.8 ± 0.7	3.2 ± 0.9	0.028
Range of motion (deg)	126.1 ± 8.6	120.8 ± 11.8	0.062

*Values are given as the mean and standard deviation*

There were significantly lower incidences of medial soft tissue release, including MCL subperiosteal elevation, MCL pie-crusting, PMC release, and SM detachment in the MFR group than in the control group. The medial release time was also significantly shorter in the MFCR group (5.1 ± 2.6 min) than in the control group (12.1 ± 4.3 min) (*P* = 0.017). Additionally, the hospital stay of the MFCR group (1.8 ± 0.7 days) was significantly shorter than that of the control group (3.2 ± 0.9 days) (*P* = 0.028). However, there was no significant difference in the range of motion between the two groups, with the MFCR group showing 126.1 ± 8.6 degrees and the control group showing 120.8 ± 11.8 degrees (*P* = 0.062). In summary, the MFCR intervention was associated with significantly reduced intraoperative blood loss, shorter operative time, shorter medial release time, and shorter hospital stay compared to the control intervention, while the range of motion did not differ significantly between the two groups.

### Functional evaluation

The comparison of WOMAC scores and walking VAS pain scores between the MFCR group (*N* = 62) and the control group (*N* = 59) is presented (Table 3). Preoperatively, there were no significant differences in any of the measured indices between the two groups. Specifically, the total WOMAC scores were 67.2 ± 9.8 in the MFCR group and 62.3 ± 16.4 in the control group (*P* = 0.2524), with similar non-significant differences observed in the pain (13.6 ± 3.4 vs. 13.1 ± 3.5, *P* = 0.2689), stiffness (4.8 ± 1.3 vs. 4.7 ± 1.8, *P* = 0.8567), and function (46.8 ± 8.1 vs. 43.2 ± 12.1, *P* = 0.6568) subscales of WOMAC.

**Table 3:** Comparison of the WOMAC Scores and Walking VAS Pain Scores

	**MFCR (*N* = 62)**	**Control (*N* = 59)**	***P*-Value**
**Preop**			
**WOMAC**			
Total	67.2 ± 9.8	62.3 ± 16.4	0.2524
Pain	13.6 ± 3.4	13.1 ± 3.5	0.2689
Stiffness	4.8 ± 1.3	4.7 ± 1.8	0.8567
Function	46.8 ± 8.1	43.2 ± 12.1	0.6568
Walking VAS pain	6.7 ± 2.2	5.9 ± 1.2	0.7526
**2 yr postop**			
**WOMAC**			
Total	29.9 ± 17.9	43.6 ± 13.7	0.0325
Pain	4.1 ± 4.7	7.7 ± 3.1	0.0238
Stiffness	3.2 ± 1.7	3.9 ± 1.7	0.0317
Function	21.5 ± 13.9	32.3 ± 11.2	0.1812
Walking VAS pain	2.1 ± 1.1	2.8 ± 1.2	0.0215

*WOMAC* = *Western Ontario and McMaster Universities Osteoarthritis Index, VAS* = *visual analog scale. The values are presented as the mean and the standard deviation*

Additionally, the walking VAS pain scores showed no significant preoperative difference (6.7 ± 2.2 vs. 5.9 ± 1.2, *P* = 0.7526) (Table 3). At 2 years postoperatively, several significant differences emerged: the total WOMAC score was lower in the MFCR group (29.9 ± 17.9) than in the control group (43.6 ± 13.7, *P* = 0.0325), with the pain subscale also showing a lower score in the MFCR group (4.1 ± 4.7 vs. 7.7 ± 3.1, *P* = 0.0238) and the stiffness subscale following the same trend (3.2 ± 1.7 vs. 3.9 ± 1.7, *P* = 0.0317) (Table 3).

In contrast, the function subscale of WOMAC did not differ significantly between the two groups at 2 years postoperatively (21.5 ± 13.9 vs. 32.3 ± 11.2, *P* = 0.1812). For walking VAS pain scores at 2 years postoperatively, the MFCR group had a lower score (2.1 ± 1.1) compared to the control group (2.8 ± 1.2, *P* = 0.0215) (Table 3).

In summary, while preoperative WOMAC scores (total and subscales) and walking VAS pain scores were comparable between the two groups, significant differences were observed at 2 years postoperatively, with the MFCR group showing lower total WOMAC pain and stiffness scores but lower walking VAS pain scores, and no significant difference in the function subscale.

### Complications

The rates of postoperative complications in the MFCR group (*N* = 62) and control group (*N* = 59) are presented (Table 4). No cases of deep periprosthetic infection, deep vein thrombosis (DVT), periprosthetic fracture, pulmonary embolism, wound complications, 30-day mortality, or calf muscular venous thrombosis were observed in the MFCR group, while the control group had 2 cases of calf muscular venous thrombosis (*P* = 0.1438). No other complications previously described were encountered.

**Table 4:** Rates of postoperative complications by group

Complication	MFCR (*N* = 62)	Control (*N* = 59)	*P*-Value
Calf muscular venous thrombosis	0	2	0.1438
Deep periprosthetic infection	0	0	-
Deep vein thrombosis	0	0	-
Periprosthetic fracture	0	0	-
Pulmonary embolism	0	0	-
PONV	2	8	0.0391
Wound complication	0	0	-
30-day mortality	0	0	-
90-day readmission	0	1	0.3033

*The values are given as a number, with the percentage in parentheses. MFCR* = *Medial Femoral Condyle Restoration, PONV* = *postoperative nausea and vomiting. Significant: P* < *0.05*

Regarding postoperative nausea and vomiting (PONV), 2 patients in the MFCR group experienced this complication, compared to 8 patients in the control group, with a statistically significant difference (*P* = 0.0391). Additionally, there were no 90-day readmissions in the MFCR group, whereas 1 patient in the control group was readmitted within 90 days (*P* = 0.3033). In summary, both groups showed a low overall incidence of postoperative complications, with no serious complications such as deep infection, DVT, or mortality observed. The only significant difference was in the rate of PONV, which was lower in the MFCR group, while other complications did not differ significantly between the two groups.

## Discussion

The most important finding of this study was that total knee arthroplasty using the MFCR technique resulted in better functional outcomes and satisfaction scores than using the control MA technique in patients with knees of varus OA. There are fewer bone resections of the femoral condyle (closer to the metal thickness of the femoral implant) and less medial soft tissue release compared to the MFCR technique to the control MA technique intraoperative.

Medial instability, including mid-flexion instability, is one of the important surgical factors that lead to abnormal kinematics, poor functional outcomes, and patient dissatisfaction [9, 15, 17, 28]. Restoring the anatomic joint line of the medial femoral condyle is the key factor to preserving the normal function of the MCL in the full range of motion. Proximal and anterior translation of the joint line had increased laxity during mid-flexion. Recutting the distal femur to elevate the joint line by 2 mm will lead to an increase of approximately 2.5° of coronal plane laxity in mid-flexion. On the other hand, 2 mm and 4 mm elevation of the joint line resulted in a 60% and 111% increase in mid-flexion laxity, respectively. In practice, the main reason for joint line elevation (over-resection of distal femoral condyle) is ignoring the cartilage loss with resection reference to the bared subchondral bone of medial femoral condyle or compensation to the over-resection of the posterior condyle to balance an enlarged flexion gap, which is often seen in the anterior reference sizer, ML/AP mismatch, or non-medial pivot posterior sizer system.

To preserve the physiological medial stability of the knee in the full range of motion, we designed the key point of the MFCR technique to restore the anatomical articulation of the medial femoral condyle. The amount of femoral bone resection is based on the true “medial condyle measured resection”. MFCR derives from the KA philosophy of restoring pre-arthritic knee morphology and the native laxity envelope while minimizing soft-tissue release, but it narrows both the reconstruction target and the boundary conditions to the anatomical restoration of the medial femoral condyle and medial stability throughout the range of motion, with predefined safety corridors for overall limb alignment and joint-line orientation; it can therefore be regarded as a constrained. Unlike KA, which pursues a comprehensive return to the individualized three-dimensional joint line and native laxity, MFCR centers on quantitatively compensating medial femoral cartilage loss and saw-kerf and prioritizing bony correction to achieve gap equality and medial stability, without intentionally reproducing or amplifying constitutional varus. As such, MFCR represents a technical compromise between restoring medial stability and avoiding excessive deviation from a near-neutral mechanical axis.

In the current study, neither navigation nor the gap balance technique was used; the intramedullary femoral cutting guide should be seated on the distal medial condyle to determine the bone cut level after compensation for the cartilage wear of the medial condyle. For the posterior condyle resection, the legion sizer was a posterior reference and a lateral pivot. The bone resection of the lateral posterior condyle was constantly 9.5 mm and equal to the implant thickness; the medial posterior condyle bone cut will increase while externally rotating relative to the posterior condyle axis. So we use a specific pinhole positioner, which is a medial pivot to guide 4 in 1 cutting block. If using the legion sizer, considering that 1° of external rotation may lead to approximately 0.8–1 mm more bone removal of the posteromedial condyle, the 4-in-1 cutting block should be moved posteriorly by 2 mm when external rotation relative to PCA was 2 degrees, and 2.5–3 mm when the external rotation was 3 degrees. So it is important for the surgeon to be familiar with the true amount of the femoral bone resection of the applied instrument and make some adjustments to fulfill the goal of medial condyle restoration. Our results showed only an average of 0.72 mm and 0.75 mm more bone of the distal medial, posterior medial femoral condyle was removed compared to the metal thickness of the implant. This was exactly the space for the cement fixation.

Another characteristic of the MFCR technique was that the bone cut orientation was adjusted based on the patient’s individual knee morphology. The target HKA was set between 0° to − 4° (− 2° ± 2°) for the varus knee instead of 0° ± 3°. The boundary of the tibial cut is in 0°–3° varus based on the patient’s individual mMPTA in a 1° compensate 4° manner. There are several advantages of the mMPTA-based adjusted tibial cut. First, the mMPTA is naturally varus, especially in the varus knee. Compared to the perpendicular cut, A slightly tibial varus cut is more natural and closer to the orientation of the natural joint line. Second, a slightly varus tibial cut may resect 1–2 mm more bone of the medial plateau than that of a perpendicular tibial cut. This can compensate for the reduced bone resection of the medial femoral condyle when the joint line is anatomically restored. Third, aiming for a perpendicular tibial cut may result in some incidence of the valgus cut, which is unnatural and may inevitably lead to excessive release of the medial soft tissue. Fourth, it is imperative to avoid excessive varus tibial cuts. Though a 5-degree varus cut was permitted in KA and its derivatives, the femoral cut is often in valgus in accordance with the femoral joint line orientation to achieve an individual HKA. In literature, more than 3° varus was related to tibial baseplate failure. The unintended excessive varus cut should be avoided when using the conventional instrument.

MFCR is not intended to preserve deliberate varus; resections are executed within a near-neutral corridor. We acknowledge that residual non-neutral alignment may theoretically increase medial compartment load, promote polyethylene edge loading, contribute to tibial radiolucency/loosening, alter joint-line obliquity, and affect patellofemoral tracking. To mitigate these risks, the protocol mandates a neutral tibial coronal cut. Any intraoperative breach of these boundaries triggers re-planning toward MA targets. While our 2-year data show no early failure signal, they cannot exclude late wear or loosening. We therefore outline a long-term surveillance program (5–10 years) assessing implant survivorship, progressive radiolucent lines, component migration, wear-related events, and PF complications, with registry linkage where available. Multicenter studies with gait/fluoroscopic contact-mechanics analyses are warranted to determine whether MFCR’s medially focused bony corrections sustain durable load distribution.

Though the definition of medial–lateral balance remains controversial, several studies have shown that the lateral side of the native knee is looser than the medial side. Total Knee arthroplasty using a medial stabilized technique that ensures medial stability and lateral laxity of the knee has also been shown to be able to restore knee medial pivot kinematics [29–31], increase postoperative knee flexion, and improve clinical outcomes. In our technique, we also aim to restore the native knee joint laxity of the lateral side. No more than 3 mm wider of the lateral gap than the medial gap under the stress test is well accepted.

The incidence of medial soft tissue release in total knee arthroplasty for varus deformity is reported to be high in MA TKA [20, 22, 32–34]. Medial soft tissue release may cause pathological lengthening of the MCL, proprioception loss, and reduced stiffness and integrity of the normal MCL. In this study, we found that the MFCR technique can significantly reduce the time and incidence of every step of medial soft tissue release. The pie-crusting release was unnecessary for all cases in the MFCR group, while this was done in the control MA group and resulted in two knees of medial instability. The effect of soft tissue preservation of the MFCR technique may be related to the equal bone resection of the distal and posterior medial femoral condyle, under correction of the varus deformity based on the patient’s individual bone morphology, as well as the concept of balance of restoring normal lateral laxity.

In this study, both the MFCR and the control MA techniques provided excellent pain relief and walking ability. However, the functional outcome of standard activities, advanced activities, as well as satisfaction scores were better in the MFCR group. We found the 13.7-point difference in WOMAC scores between groups. We anchored interpretation to published MCID thresholds for TKA: anchor-based studies suggest an MCID of 10–15 points for WOMAC total, and systematic reviews report comparable ranges across WOMAC subscales. Thus, our observed mean difference meets or exceeds commonly accepted clinical relevance.

The lower PONV rate observed in the MFCR cohort is plausibly explained by several mechanistic features of the technique. The shorter balancing and operative times may reduce exposure to inhalational agents and other emetogenic anesthetics. And the bony-first, minimal-release strategy likely decreases nociceptive input and tissue trauma, permitting lower perioperative opioid requirements; and improved early comfort may facilitate earlier mobilization and oral intake, both protective against PONV. While our trial was not designed to confirm these mediating pathways, these hypotheses are biologically credible and consistent with perioperative physiology. Future studies should standardize anesthesia/analgesia protocols and prospectively capture opioid consumption, antiemetic use, and time to oral intake to test whether the MFCR-related workflow changes causally reduce PONV.

The flexion gap balance technique aims to restore the medial joint line [35–37]. Besides, peak quadriceps force was significantly higher compared to traditional gap-balancing techniques. More physiological knee kinematics with greater lateral femoral rollback were achieved using a modified technique to restore the normal medial joint line. Restoration of constitutional alignment during TKA leads to more physiological peri-articular soft tissue strains [38–40]. We hypothesized that restoring the medial femoral condyle anatomy while preserving slight varus alignment based on individual knee morphology, reestablishing normal knee laxity, and protecting medial soft tissues could collectively achieve: A medially stable knee throughout the full range of motion, optimized quadriceps function, medial pivot kinematics, and more natural knee proprioception. This may account for the better function and satisfaction scores in the patients who underwent TKA with the MFCR technique.

## Limitations

This study has the following limitations. First, the study population was restricted to patients with varus knee osteoarthritis, which may limit the generalizability of the findings to other types of knee deformities, such as valgus osteoarthritis. Second, surgeries were performed using conventional manual instruments, which inevitably introduced potential errors in bone resection accuracy, soft tissue balancing, and achievement of individual alignment targets. This study did not include preoperative quantitative cartilage imaging (e.g., MRI 3D-DESS); therefore, the estimation of medial cartilage loss relied on intraoperative visual grading with calibrated probing and a rule-based millimeter conversion. Although simple and pragmatic, this approach is inherently subjective and may under- or over-estimate true cartilage thickness, especially in focal posterior lesions. Future studies should validate this grade-to-millimeter conversion against quantitative imaging, and utilize advanced navigation or robot-assisted systems could enhance procedural precision and reduce such variability. Third, while we managed multiplicity through a predefined endpoint hierarchy, comprehensive correction of all secondary endpoints may be overly conservative. These exploratory findings should be interpreted cautiously, with focus on effect sizes and confidence intervals rather than *P*-values alone. Fourth, the follow-up duration for clinical outcomes remains relatively short, and the study was conducted at a single center, which may affect the external validity of the results. Longer-term follow-up is necessary to evaluate the long-term efficacy and prosthesis survival, particularly given the slight varus position of the tibial component, and multi-center studies would help verify the generalizability of the findings.

## Conclusion

MFCR achieved anatomical restoration of the medial femoral condyle in varus TKA, lowered perioperative burden, and improved early functional recovery relative to mechanical alignment through a standardized, millimeter-level, bony-first, minimal-release workflow that does not rely on advanced imaging or robotics.

## Data Availability

All data and materials are available on reasonable request.
